# New Alternative Techniques for Sentinel Lymph Node Biopsy

**DOI:** 10.3390/medicina59122077

**Published:** 2023-11-26

**Authors:** Subiksha Subramonian, Sharat Chopra, Raghavan Vidya

**Affiliations:** 1 Royal London Hospital, London E1 1FR, UK; 2 Aneurin Bevan University Health Board, The Royal Gwent Hospital, Newport NP20 2UB, UK; sharat.chopra2@wales.nhs.uk; 3 The Royal Wolverhampton NHS Trust, Wolverhampton WV10 0QP, UK

**Keywords:** sentinel lymph node, biopsy, breast cancer, techniques, axilla, localisation

## Abstract

*Background and Objectives*: This review paper highlights the key alternatives to the blue dye/radioisotope method of sentinel lymph node biopsy (SLNB). It analyses the research available on these alternative methods and their outcomes compared to the traditional techniques. *Materials and Methods*: This review focused on fifteen articles, of which five used indocyanine green (ICG) as a tracer, four used magnetic tracers, one used one-step nucleic acid amplification (OSNA) and Metasin (quantitative reverse transcriptase-polymerase chain reaction), one used the photosensitiser talaporfin sodium, one used sulphur hexafluoride gas microbubbles, one used CT-guided lymphography and two focused on general SLNB technique reviews. *Results*: Of the 15 papers analysed, the sentinel node detection rates were 69–100% for indocyanine green, 91.67–100% for magnetic tracers, 81% for talaporfin sodium, 9.3–55.2% for sulphur hexafluoride gas microbubbles, 90.5% for CTLG and 82.7–100% for one-step nucleic acid amplification. *Conclusions*: Indocyanine green fluorescence (ICG) and magnetic tracers have been proven non-inferior to traditional blue dye and isotope regarding SLNB localisation. Further studies are needed to investigate the use of these techniques in conjunction with each other and the possible use of language learning models. Dedicated studies are required to assess cost efficacy and longer-term outcomes.

## 1. Introduction

Breast cancer is the most common cancer in the United Kingdom, with one woman diagnosed every 10 min [1]. Axillary node management has long been a controversial question for breast surgeons nationwide, with a shift in strategy from the most aggressive but tolerable treatment to the minimal effective treatment. Sentinel lymph node biopsy (SLNB) was proven non-inferior to axillary node dissection [2], and SLNB has been the gold standard for axillary staging of breast cancer since its first use in 1994 [3].

The two most commonly used methods for SLNB are blue dye and radioisotope (RI) labelling, with a combination of blue dye/RI giving the most accurate results. However, these methods come with significant disadvantages, including cost, risk of an allergic reaction, the need for a nuclear medicine department and scheduling of isotope injections preoperatively. In some countries, such as Japan, the lack of nuclear medicine facilities [4] has led to the development of alternative methods of SLNB. This review analyses the alternatives available, including their localisation outcomes, advantages and disadvantages.

## 2. Materials and Methods

We searched the PubMed database for articles published between 1 January 2013 and 30 June 2023 using the following search term: ‘alternatives sentinel lymph node biopsy breast cancer’. This yielded 548 results. Inclusion criteria filters were applied, including:

- Date of publication 1 January 2013 to 30 June 2023, narrowing the list to 242 results.
- Language of publication English only, further narrowing the list to 229 results.
- Article types filtered to only include clinical trials, randomised controlled trials, reviews and systematic reviews, narrowing the list to 59 results.
- Finally, a ‘full text’ filter was applied, which yielded a final 58 articles.

**Figure 1:**
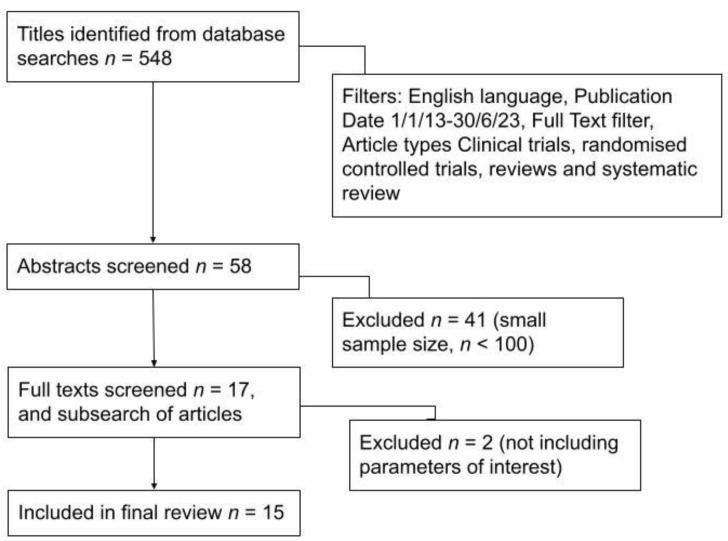
PRISMA diagram showing literature search process.

Fifty-eight article abstracts were screened. Forty-one articles were excluded, as they did not focus on our parameters of interest—specifically techniques used to identify the sentinel node in breast cancer, SLNB outcomes and the advantages and disadvantages of these methods. Irrelevant articles were excluded, such as those focusing on non-breast cancers, sentinel node biopsy vs axillary node dissection, or wireless vs. guided localisations. Thus, seventeen final articles were screened, of which ten were excluded as they had a small sample size (less than 100 patients), yielding seven final articles. Subsearching these articles yielded eight more articles that were deemed relevant to this paper (focusing on sentinel node biopsy techniques, advantages and disadvantages) and of a sufficient sample size (*n* > 100) to draw accurate conclusions. Thus, fifteen articles were finally included in this review paper, consisting of five on indocyanine green (ICG) fluorescence, four on magnetic tracers, four on other methods (photosensitiser, contrast ultrasound gas microbubbles, nucleic acid amplification and CT lymphography) and, finally, two review papers on SLNB techniques. The PRISMA diagram in Figure 1 describes the literature search process.

The strengths of our paper include being one of the first review papers to compare all of the alternatives to the sentinel lymph node biopsy in breast cancer treatment, as a search on PubMed with the term ‘Alternative sentinel lymph node biopsy breast cancer’ revealed that there are currently no review papers which do this. The limitations of our study are that we did not undertake a statistical pooled analysis to formally compare the sentinel node outcomes across each paper. Furthermore, there are limited data available on the long-term effects of these new technologies (e.g., regional recurrence and overall survival rate) and the conclusions we can draw about long-term safety, efficacy and future consequences are thus limited. There is also a control bias as methods were not tested in isolation—e.g., most papers assessed the new localisation technology with radioisotope and/or blue dye as concurrent controls.

## 3. Results

The various methods analysed in this review are described below.

### 3.1. Indocyanine Green (ICG) Fluorescence

ICG is a low-molecular-weight, non-toxic, non-radioactive substance with a short serum half-life [5]. It is injected into the subdermal/peritumoral region just before surgery, which enables visualisation of the lymphatic drainage to the axilla using a fluorescence probe.

SLNB outcomes for ICG are shown in Table 1 below.

### 3.2. Magnetic Tracers, Most Commonly SPIO (Superparamagnetic Iron Oxide)

A magnetic tracer is injected into the breast, and the sentinel node is stained brown and identified with a handheld magnetometer. The magnetic tracers studied in this review [10] include ferumoxytol, magnetite/maghemite and ferumoxide.

SLNB outcomes for magnetic tracers are shown in Table 2 below.

### 3.3. Photosensitiser Talaporfin Sodium

Talaporfin is injected at the subareolar region just before the operation, and the sentinel node is identified as ‘pink’ by using fluorescence with xenon light [4].

### 3.4. Contrast-Enhanced Microbubbles

An ultrasound contrast agent (sulphur hexafluoride gas microbubbles) is injected at the periareolar region [14], followed by ultrasound imaging of the breast, with immediate visualisation of the lymphatic channels towards the sentinel node, which is then marked with a clip/wire.

### 3.5. One-Step Nucleic Acid Amplification (OSNA)/Reverse Transcriptase Gene Therapy (Metasin)

The OSNA test analyses genetic material from solubilised SLNB biopsy samples [15] and detects the presence of the cytokeratin-19 (CK19) gene, a marker associated with breast cancer—and will provide a result within a short time, enabling surgeons to determine whether other lymph nodes should be removed at the same time. The Metasin test uses the quantitative reverse transcriptase-polymerase chain reaction (qRT-PCR) to detect CK19 and mammaglobin (two markers of metastases).

### 3.6. Computed Tomography Lymphography (CTLG)

CT-guided lymphography (CTLG) involves the injection of iopamidol (CT dye) into the subareolar region [16], followed by contiguous CT imaging to identify the sentinel node (the node that enhances with the dye). The location of the sentinel node can be mapped on the skin by marking the crossing point of the lines of the laser beam of the CT.

SLNB outcomes for these four methods are displayed below in Table 3.

As Table 1, Table 2 and Table 3 demonstrate, the SLNB detection rates for ICG fluorescence and magnetic tracers are generally higher than those of the other methods.

Table 4 below illustrates each method’s main advantages and disadvantages, as taken from each paper.

## 4. Discussion

### 4.1. Discussion of Techniques Evaluated in this Paper

Accurate sentinel lymph node assessment is vital in managing breast cancer. As the Z11 trial [2] has shown, sentinel lymph node biopsy (SLNB) has shown to be non-inferior to axillary node dissection. It offers the added advantage of reduced morbidity and reduced side effects such as axillary lymphedema, numbness and reduced range of motion. The standard method of SLNB has thus far been dual: radioisotope and blue dye injection [18]. However, many countries worldwide do not have nuclear medicine facilities [4], and the need for preoperative radioisotope injections creates scheduling difficulties and the need for safe handling and disposal of these materials. Blue dye has been used for many years as a localisation technique, but it can cause skin staining for months to years and poses a potential allergy risk to patients. There are many up-and-coming alternative techniques for SLNB, and this paper reviewed the most common methods.

Indocyanine green (ICG) fluorescence is one of the most well-known alternative methods for SLNB localisation. As Table 1; Table 4 demonstrate, ICG fluorescence offers a comparable SLNB detection rate to radioisotope/blue dye [8], and it has not shown any allergic reactions in the studied patient samples as compared to blue dye. ICG is a non-toxic substance with a low molecular weight, allowing it to move freely through vessels damaged by neoadjuvant chemotherapy, surgery or radiotherapy-related fibrosis. The evidence suggests SLNB via ICG fluorescence is up to five times less expensive than that of a radioisotope [8], and ICG-guided SLNB does not increase operative time compared to traditional methods. However, ICG has difficulty penetrating deeper tissues (beyond 10–20 mm), giving it the same difficulties as blue dye/radioisotope in patients with a high BMI [7]. ICG leakage is one of its main drawbacks, as it can be challenging to localise damaged nodes if ICG leakage has caused vessel rupture and contamination of the surroundings with ICG. As with many new SLNB methods, ICG fluorescence also increases the number of lymph nodes that are removed. However, there is no clear analysis of whether this leads to increased patient morbidity; thus, more detailed follow-up studies are needed. Furthermore, there are not many widespread data on the cost efficacy of ICG.

Magnetic tracers offer a tangible alternative to SLNB detection methods. The size of the magnetic particles used hugely influences the outcome, with the medium-sized tracer (59 mm) demonstrated to be the most accurate at SLNB localisation [10]. More data have been published on the relative costs of magnetic tracers in SLNB localisation when compared to other new techniques. The mean cost for superparamagnetic iron oxide (SPIO) was EUR 225, compared with EUR 252 for the radioisotope [11]. The preoperative administration of SPIO saved at least 20 min of operating theatre time, saving an additional EUR 352.70 per procedure. The smallest tracer size (32 mm) is disadvantageous as it passes through the sentinel node and up to higher-level nodes, increasing the number of nodes removed, with subsequent possible morbidity [10]. However, magnetic residue was found to last in tissue up to at least 42 months postoperatively, causing artefacts on MRI scans in 40% of cases [17]. Further work has been carried out on SPIO magnetic tracers, including use of an ultra-low dose of 0.1 mL SPIO injections (compared to the usual 1–2 mL dose), achieving a 100% SLN detection rate with reduced skin staining [19]. However, further studies are needed to prove the long-term efficacy of this ultra-low dose, with follow-up imaging to prove a reduction in artefacts on MRI scans.

Other methods of SLNB detection include a photosensitiser (talaporfin), which was able to identify sentinel nodes of all patients [4] but did not show a consistent correlation with fluorescence and pathological node metastases. A more novel SLNB technique includes a contrast-enhanced ultrasound (CEUS), with hexafluoride gas microbubbles. This method caused less trauma to the lymph nodes [14] and was able to localise the SLN accurately, with an overall detection rate of 92.8% in a recent meta-analysis [18]. However, the false negative rate of CEUS is higher than that of radio tracer alone, though lower than that of blue dye [18]. The contrast used for CEUS is not specifically designed for SLNB and the size of the microbubble particle can cause problems with the accuracy of the method. Moreover, there is a minimal number of single-centre studies assessing this technique; thus more data are needed to assess its efficacy and safety.

One-step nucleic acid amplification (OSNA) has also been trialled in SLNB, screening for CK19 expression from breast tumour cells [15]. The test provides results within 30 min, enabling intra-operative decisions about further lymph node removal. OSNA has also been trialled in the treatment of other cancers, and has been shown to detect a higher percentage of micrometastatic lymph nodes in patients with non-small-cell lung cancer (NSCLC) and colorectal carcinoma (CRC) [20]. However, the higher sensitivity of OSNA does not correlate with disease progression when compared to traditional haematoxylin and eosin (H&E) staining. The data have also shown that OSNA is not cost-effective [15] for SLNB compared to histopathology as a standard method of cell analysis, and some tumour cells do not express CK19 MRA and thus will not be detected [15]. Finally, CT lymphography (CTLG) offers a novel way of localising the sentinel node [16], with possible combination with other techniques (e.g., ICG fluorescence) allowing for a non-invasive, highly accurate localisation process when compared to the radioisotope/blue dye method.

### 4.2. Future Directions for Sentinel Node Biopsy in Breast Cancer

The current standard of care in breast cancer is for patients with positive sentinel lymph nodes to progress to completion of axillary node dissection—a procedure with a high morbidity risk. The mandate for future breast surgery techniques is to provide the most clinically effective care with the least invasive/harmful methods. This is paralleled in other specialties, such as in the management of ovarian cancer, where ongoing research is being conducted into sentinel node staging [21] to enable minimally invasive surgery and staging of nodal status, as an alternative to open laparotomy with total hysterectomy, bilateral salpingo-oophorectomy, lymphadenectomy and omentectomy.

The current practice in SLNB diagnosis involves the biopsy samples being sent for histopathological assessment, which is a time-consuming process with less-than-ideal sensitivity and specificity. New studies have looked at the possible use of artificial intelligence algorithms (AI) in reducing the workload of pathologists, enabling faster and more accurate pathological outcomes. Holten-Rossing et al. [22] conducted a 135-patient digital image analysis study where sentinel nodes were assessed by pathologists conventionally, and simultaneously the stained sections were digitally analysed by an algorithm (which was trained to assess for cytokeratin antibodies and staining intensity). The digital algorithm had an SLNB sensitivity of 100% with no false negative results, and across the three centres it could have decreased the workload by 58.2% on average [22]. The study also concluded that pathologists spend an average of 6.88 min per sentinel node, and if 58.2% of these samples are excluded from conventional microscopy because of digital algorithm pre-screening, this could save up to five working days of time (based on an average of 580 patients annually) for pathologists [22]. However, this small time study only focused on 12 patients and assumed that the time taken to assess positive and negative nodes would be the same. The same idea has also been explored in radiology. Ha et al. [23] evaluated the role of the conventional neural network (CNN) in examining breast MRIs and predicting lymph node metastasis. In this study, with 133 metastatic axillary nodes and 142 negative control nodes, the CNN was able to identify metastatic nodes with an accuracy of 84.3% [23], showing that with further training on larger datasets, these CNN models may eventually be able to remove the need for core needle or sentinel lymph node biopsies.

Using language learning models in conjunction with new SLNB techniques could offer a promising future for breast cancer surgery, driving a decrease in invasive axillary dissection surgeries. In light of the need for less invasive procedures, there is a keen interest in studies that analyse which breast cancer patients with positive sentinel nodes can safely have axillary node dissection omitted. Wu et al. [24] highlight an interesting point about the possible future use of language learning models in breast cancer management and SLNB. They use a predictive language learning model, focusing on four key factors (number of positive SLNs, total number of SLNs harvested, absence of hilar lymph node, and lympho-vascular invasion) to generate a predictive nomogram to identify patients that could safely avoid axillary node dissection [24].

The future of sentinel node biopsy in breast cancer is moving towards a hybrid approach with multimodal techniques [18] and concurrent real-time visualisation of the lymphatic drainage pathways. The current gold standard for SLNB is a combined radioisotope and blue dye method to improve detection rates and reduce false negative rates. Mokhtar et al. [25] analysed the use of a new triple protocol, consisting of preoperative CT lymphography, intraoperative SLNB with ICG fluorescence and intraoperative one-step nucleic acid amplification (OSNA) to detect positive nodes. OSNA when compared to the traditional histopathological analysis showed a concordance of 90% [25], thus allowing the completion of axillary surgery in one combined procedure, rather than needing a second procedure, leading to reduced costs and better patient outcomes [25].

## 5. Conclusions

Indocyanine green fluorescence (ICG) and magnetic tracers have been proven non-inferior to traditional blue dye and isotope regarding SLNB localisation. Further work is needed to assess the long-term effects of these techniques, particularly the use of magnetic tracers and postoperative MRI scans. More recent techniques include nucleic acid amplification, which has not yet been tested on a large human population. These therapies offer promising SLNB localisation techniques, particularly in conjunction with new language learning models and digital algorithm screening, for the possible omittance of axillary dissection surgery. As with any new methods, there would be a retraining period where surgeons would have to acclimate to the new technique. However, many of these methods are similar to the radioisotope/blue dye technique, using slightly different materials. Further research is needed to assess the longer-term effects of these new techniques in isolation (rather than combined studies using new techniques with blue dye/isotope as controls), and dedicated studies into cost savings are required.

## Figures and Tables

**Table 1:** SLNB outcomes for Indocyanine green (ICG) fluorescence.

Paper Author, Year.	SLNB Detection Rate with ICG Dye (Compared to Alternative)
Yamamoto S, 2013 [6]	99.6% (90.3% blue dye)
Vaz T, 2018 [5]	91.4% (89.5% blue dye, 97.2% radioisotope)
Bargon CA, 2022 [7]	96.1% (86.4% radioisotope)
Thongvitokomarn S, 2020 [8]	69–100% (85–100% radioisotope)
Goonawardena J, 2020 [9]	81.9–199% (85–199% radioisotope)

**Table 2:** SLNB outcomes for magnetic tracers.

Paper Author, Year	SLNB Detection Rate with Magnetic Tracer (Compared to Alternative)
Pouw J, 2015 [10]	100% retrieval rate Ferumoxytol/Sienna. 91.67% ferumoxide (alternative not reported)
Karakatsanis A, 2017 [11]	95.6% SPIO (96.6% radioisotope)
Man V, 2019 [12]	98.8% SPIO (alternative not reported)
Thompson W, 2020 [13]	99.3% SPIO (98.6% radioisotope)

**Table 3:** SLNB outcomes for other new techniques.

Paper Author, Year	SLNB Detection Rate with New Techniques (Compared to Alternative)
Yamada K, 2013 [4]	81% talaporfin sodium (86% radioisotope)
Gkegkes ID, 2015 [14]	9.3–55.2% contrast-enhanced ultrasound (CEU) sulphur hexafluoride gas microbubbles (alternative not reported)
Huxley N, 2015 [15]	82.7–100% for one-step nucleic acid amplification (alternative not reported)
Hamdy O, 2021 [16]	90.5% CTLG (alternative not reported)

**Table 4:** Comparison of the alternative techniques.

Technique	Type of Tracer	Publication	Sample Size	Advantages	Disadvantages
Indocyanine green fluorescence (ICG) [6]	Indocyanine green (ICG), blue dye as control	Japan, 2013	258 women	-ICG lasts 3 h post-injection, and thus can be injected outside the operating room -RVS detects metastatic nodes preoperatively so they can proceed directly to axillary dissection	-Injured lymph vessels cause ICG field contamination -Operating light needs to be turned off during fluorescence observation
Indocyanine green fluorescence (ICG) [5]	Indocyanine green (ICG)	Portugal, 2018	232 patients	-ICG is low-molecular-weight, non-toxic and non-radioactive -ICG’s low molecular weight and albumin binding aids SLN detection in neoadjuvant patients, as ICG can move through vessels obliterated by tumour cells/inflammation/previous surgery/RT-related fibrosis -Low cost of ICG	-ICG low tissue penetration (10–20 mm depth) -Need for a photodynamic chamber with ICG -Increased number of fluorescent lymph nodes removed and subsequent morbidity
Indocyanine green fluorescence (ICG) [7]	Indocyanine green (ICG)	Amsterdam, 2022	102 patients	-The ICG method did not increase detection/operative time -ICG is non-ionising and does not need special storage, handling procedures or legal permits -ICG fluorescence is five times less expensive than nanocolloid, and the fluorescent camera can be used for other indications	-ICG use for axillary SLNB has not yet been approved by the US FDA and the European Medicines Evaluation Agency (EMEA) -Difficult SLNB detection in high-BMI patients
Indocyanine green fluorescence (ICG) [8]	Indocyanine green (ICG)	Thailand, 2020	4216 SLNB procedures	-ICG has a short half-life and is absorbed into lymphatic vessels immediately -ICG is potentially available for use in pregnant women, with some human and animal studies	-ICG not visualised directly, and difficulty identifying SLNs from the screen -Insufficient data regarding long-term follow-up for ICG-SLNB
Indocyanine green fluorescence (ICG) [9]	Indocyanine green (ICG)	Australia, 2020	2301 patients	-Dual mapping with ICG + RI decreased false negative rate < 8.7% (compared to RI + BD) -ICG method costs USD 5–111 per patient (RI costs USD 331–420 per pt)	-Optimal ICG dose/concentration not yet standardised -ICG cannot be given to patients with iodine allergy
Magnetic tracer [10]	1. Feraheme^®^ (ferumoxytol) 2. Sienna+^®^ (Fe/m) 3. Endorem^®^ (ferumoxide)	London, 2015	18 mini-pigs	-59 mm tracer (Sienna^®^) is the best-performing tracer approved for human use in SLNB -Node localised within 30 min -Feraheme^®^ and Sienna^®^ tracer 100% retrieval rate (12/12 magnetic hotspots), ferumoxide retrieval rate 91.67% (11/12 magnetic hotspots)	-Skin discolouration at the injection site -Significantly higher number of nodes excised with 32 nm tracer as the small particle passes onto higher-level nodes
Magnetic tracer [11]	Superparamagnetic iron oxide (SPIO) nanoparticles	Sweden, 2017	338 patients	-SPIO costs EUR 225 and radioisotope costs EUR 252, with SPIO preoperative administration saving 20 min operating room time, further saving EUR 352.70 -SPIO resides in tissue for a prolonged period, so operation rescheduling does not require another injection, providing flexibility -SPIO simple administration only needs a portable probe and a room-temperature tracer	-Patients received injections of SPIO and radioisotope, and there was synergy bias -Skin staining in 39.9% of patients, with 41.2% still stained after 12 months -SPIO causes artefact on MRI, even in postop scans in the future
Magnetic tracer [12]	Superparamagnetic iron oxide (SPIO)	Hong Kong, 2019	328 females	-Outpatient clinic SPIO offers better preoperative logistics -No reported adverse reactions -SPIO alone in SLNB localisation saves an estimated USD 22,300 per year compared to conventional dual tracers	-Plastic operating instruments are required to avoid interference with the magnetometer -Persistent brown skin discolouration, and MRI scan artefact for up to 1.5 years
Magnetic tracer [13]	Superparamagnetic iron oxide (SPIO)	Canada, 2020	1834 patients	-Detection rate for SPIO was non-inferior to RI method -Magnetic localisation systems found to be generally safe (with minor skin staining) -Magnetic localisation was USD 27 cheaper than radioisotope method (USD 225 for magnetic localisation, USD 252 for RI)	-No guidelines available on use of magnetic localisation in sentinel node biopsy -No data on false positive rates (which would be helpful for avoiding unnecessary procedures and influencing policy decisions)
Photosensitiser talaporfin sodium [4]	Photosensitiser talaporfin sodium	Japan, 2013	21 women	-Talaporfin is metabolised in the liver, so is safe for renal failure patients -No adverse effects of talaporfin -Talaporfin identified SLNs and also the pathways to secondary SLNs	-No consistent correlation between fluorescence and pathological SLN metastasis -The amount of radioisotope and depth of injection may affect SLN identification
Contrast-enhanced ultrasound (CEU) using sulphur hexafluoride gas microbubbles [14]	Sulphur hexafluoride gas microbubbles	Greece, 2015	727 patients	-CEU method causes less trauma and architectural disruption to the lymph nodes’ precise sampling -The CEU method duration is roughly 25 min and does not cause discomfort to the patient—no published complications of this method so far	-Limited number of studies and small patient sample size—majority of existing evidence is from one centre
One-step nucleic acid amplification (OSNA)/ reverse transcriptase gene therapy (Metasin) [15]		UK, 2015	4604 patients 18 studies	-OSNA provides results within 30–45 min and can be used during breast surgery to determine if other lymph nodes should be removed at the same time -OSNA does not require mRNA extraction/purification from the tissue -Metasin test takes roughly 30 min in total for results	-OSNA QALY loss of 0.048 relative to histopathology, not cost-effective and less accurate -1% of breast tumours do not express CK19 MRA -Entire node analysis required as tumour cells may not be evenly distributed through the node
CT lymphography [16]	Iopamidol	Egypt, 2021	835 patients	-CTLG 92.6% sensitivity, 88.6% specificity—potentially possible to omit SLN biopsy -CTLG is unaffected by lymph node fibrosis after neoadjuvant (90.5% detection rate post neoadjuvant)	-Further studies are needed to evaluate the use of CTLG in isolation compared to radioisotopes alone (rather than combination studies which introduce bias)
Review paper [17]		Switzerland, 2018	24 patients	-In 48% of the imaging cases, there was no restriction of MRI post magnetic tracer (average time since tracer injection was 42 months)	-40% impaired imaging, and 12% MRI impossible due to Sienna tracer residue -Sienna tracer causes skin discolouration in 19–40% of patients and persists in 8.6% after 15 months
Review paper [18]		Italy, 2020	Not reported	-Multimodal and hybrid techniques (preoperative CTLG + intraoperative SLNB with fluorescence navigation + OSNA) are an attractive option for institutions without nuclear medicine facilities and may help omit need for 2nd procedure in positive SLN patients	-Legislation problems with all new/hybrid techniques -Needs evaluation in larger, multi-centre studies

## Data Availability

No new data were created or analyzed in this study. Data sharing is not applicable to this article.
